# Three-Dimensional Photon Counting Imaging with Axially Distributed Sensing

**DOI:** 10.3390/s16081184

**Published:** 2016-07-28

**Authors:** Myungjin Cho, Bahram Javidi

**Affiliations:** 1 Department of Electrical, Electronic, and Control Engineering, Institute of Information Telecommunication Convergence (IITC), Hankyong National University, 327 Chungang-ro, Anseong-si, Kyonggi-do 456-749, Korea; 2 Electrical and Computer Engineering Department, University of Connecticut, Unit 4157, Storrs, CT 06269, USA; bahram@engr.uconn.edu

**Keywords:** axially distributed sensing, photon counting imaging, statistical estimation, Poisson distribution

## Abstract

In this paper, we review three-dimensional (3D) photon counting imaging with axially distributed sensing. Under severely photon-starved conditions, we have proposed various imaging and algorithmic approaches to reconstruct a scene in 3D, which are not possible by using conventional imaging system due to lack of sufficient number of photons. In this paper, we present an overview of optical sensing and imaging system along with dedicated algorithms for reconstructing 3D scenes by photon counting axially distributed sensing, which may be implemented by moving a single image sensor along its optical axis. To visualize the 3D image, statistical estimation methods and computational reconstruction of axially distributed sensing is applied.

## 1. Introduction

Under severely photon-starved conditions, scenes recorded optically may not be properly reconstructed by conventional imaging systems. In many fields, such as noninvasive microscopy, night vision, astronomy, military applications, etc., image acquisition or visualization may be carried out in a low light level environment. Recently, many approaches for three-dimensional (3D) photon counting imaging have been reported [1,2,3,4,5,6]. For example, 3D information can be recorded and reconstructed under photon-starved conditions with photon counting integral imaging [7,8,9,10,11,12,13]. In 3D photon counting imaging, 3D images can be visualized by statistical estimations, such as maximum likelihood estimation [2] and Bayesian approaches [4]. Photon counting detection under such conditions can be modeled using a Poisson distribution since photon events may occur rarely in unit time and space [14]. Using this mathematical photon counting imaging model, 3D visualization and object recognition can be performed under photon-starved conditions. Additionally, optical encryption with improved security level has been accomplished photon counting imaging properties [15,16,17].

In photon counting imaging, the visual quality of the recorded image or the reconstructed image depends on the number of photons from the scene. To enhance its visual quality, some techniques have been proposed [2,3,4]. Three-dimensional photon counting imaging captures multiple 2D images from the scenes using a lenslet array or moving camera. The statistical properties of the optical rays, as well as photon counting, are used and the visual quality of the reconstructed image can be enhanced. 

To obtain the 3D information with high resolution from the scenes and remove the requirement of lateral parallax of image sensor by integral imaging [11], axially distributed sensing (ADS) may be used [12]. In order to obtain the 3D information, ADS uses only a single camera moving along its optical axis. We show that photon counting with ADS [5] can be used to obtain the 3D information of the scenes under photon-starved conditions. We can obtain the depth map of the 3D objects. Thus, we can create the 3D profile of the object and regenerate elemental images for multiple viewing points for 3D display [18,19,20,21].

In this paper, we present an overview of the basic concept of photon counting imaging, and our work on 3D reconstruction using 3D photon counting ADS along with some experiments to illustrate 3D photon counting imaging with ADS.

## 2. 3D Photon Counting Imaging

### 2.1. Mathematical Model of Photon Counting Detection

**Figure 1:**
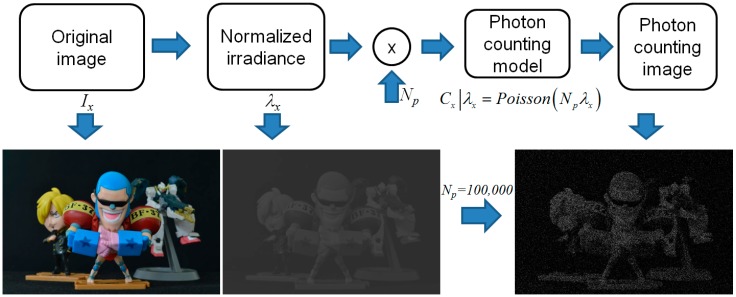
Mathematical model of photon counting detector.

Photon counting detection may be modeled by Poisson distribution because the photon events occur rarely in unit time and space under photon-starved conditions [14]. The photon counting detection fundamental steps are illustrated in Figure 1. For computational simplicity, the image has only one-dimension. Using the following equation, the photon counting image can be constructed [2].

(1) $$\lambda_{x}=\frac{I_{x}}{\sum_{x=1}^{N_{x}}I_{x}}$$

(2) $$C_{x}|\lambda_{x}\sim Poisson\left(N_{p}\lambda_{x}\right)$$

where *I_x_* is the light intensity of the image at pixel *x*, *N_x_* is the total number of pixels in the image, *λ_x_* is the normalized irradiance at pixel *x*, *N_p_* is the extracted number of photons from the image, *C_x_* is the number of photons at pixel *x*, respectively. Here, the total energy of *λ_x_* is unity.

Now, we have 2D photon-limited images, which are generated from Equations (1) and (2). When *N_p_* is very small or the scenes are under severely photon-starved conditions, the image cannot be visualized or recognized. We have shown that passive 3D imaging technique such as integral imaging [1,2,3,4] can enhance the visual quality of these photon-limited images and obtain the 3D information. In integral imaging, 3D information can be recorded through a lenslet array or a camera array. Here, multiple 2D images with different perspectives can be acquired. These images are referred to as elemental images. To obtain high lateral and depth resolutions, synthetic aperture integral imaging (SAII) [11], which uses multiple cameras can be used. Then, using computational integral imaging reconstruction (CIIR) [13], 3D images with enhanced visual quality can be reconstructed. In 3D photon counting integral imaging, to estimate 3D information from multiple photon-limited images, statistical estimations such as maximum likelihood estimation (MLE) [2] may be used, as well as computational reconstruction [13]. 

### 2.2. Photon Counting Axially Distributed Sensing (ADS)

**Figure 2:**
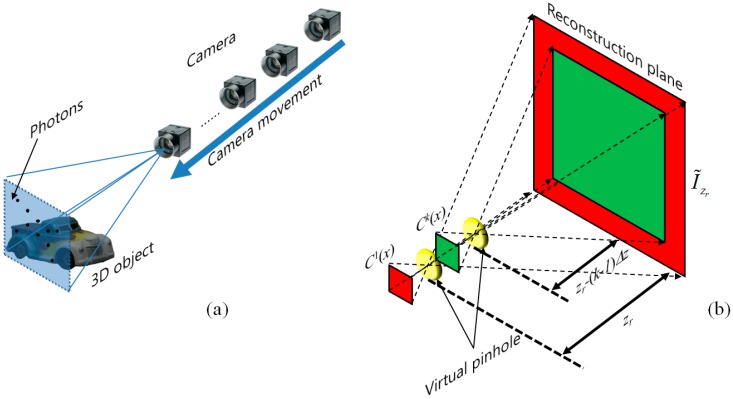
Photon counting imaging with ADS. (**a**) Image sensing; (**b**) Computational reconstruction, k is the number of recorded images by ADS.

To remove lateral movement of image sensor required by SAII, axially distributed sensing has been reported [12]. It can capture multiple 2D images with slightly different perspectives by moving single image sensor along its optical axis. Thus, we can adopt this technique to 3D photon counting imaging. Figure 2 shows the basic concept of 3D photon counting imaging with ADS for pickup and reconstruction. 

In the pickup process, by moving single photon counting camera along its optical axis, multiple 2D images with slightly different perspectives can be recorded as shown in Figure 2a. Computational reconstruction of ADS can be implemented by considering slightly different magnification ratio for each photon-limited image as the following equation [5]:

(3) $$M_{k}\left(z_{r}\right)=\frac{z_{r}-k\Delta z}{z_{r}}$$

where Δ*z* is the moving step for single camera along its optical axis, *z_r_* is the reconstruction depth, and *k* is the index of the recorded images by ADS, respectively. Since magnification causes the degradation of the reconstructed 3D image quality by the image interpolation method, in this paper, demagnification is used. Then, using MLE process [2], computational reconstruction of photon counting ADS as shown in Figure 2b can be implemented by follows [5]:

(4) $$L\left(N_{p}\lambda_{k}|C_{k}\right)=\prod_{k=1}^{K}\frac{{\left(N_{p}\lambda_{k}\right)}^{C_{k}}e^{-N_{p}\lambda_{k}}}{C_{k}!}$$

(5) $$l\left(N_{p}\lambda_{k}|C_{k}\right)\propto \sum_{k=1}^{K}\left[C_{k}\log\left(N_{p}\lambda_{k}\right)-N_{p}\lambda_{k}\right]$$

(6) $$\frac{\partial \left(N_{p}\lambda_{k}|C_{k}\right)}{\partial \lambda_{k}}=0,\;\;{\hat{\lambda }}_{k}=\frac{C_{k}}{N_{p}}$$

(7) $$\hat{I}{\left(x,z_{r}\right)}_{ADS}=\frac{1}{N_{p}K}\sum_{k=1}^{K}C_{k}\left(\frac{x}{M_{k}\left(z_{r}\right)}\right)$$

where *L*(·|·) and *l*(·|·) are the likelihood function and log-likelihood function, *C_k_* is the *k*th photon-limited elemental image by ADS and *K* is the total number of the captured photon-limited elemental images, respectively.

## 3. Experimental Results

### 3.1. Photon Counting Imaging with Axially Distributed Sensing

#### 3.1.1. Experimental Setup

**Figure 3:**
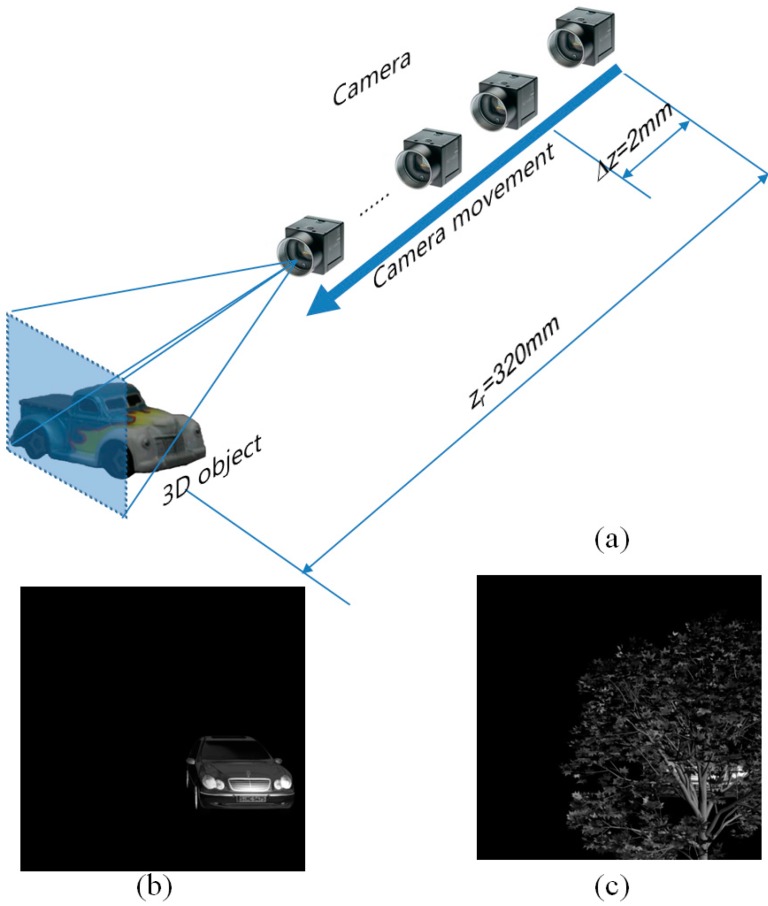
(**a**) Experimental setup for ADS; (**b**) Non-occluded 3D object; (**c**) Partially occluded 3D object.

Experimental setup for photon counting ADS is illustrated in Figure 3a. In this setup, the focal length of the camera lens is 50 mm. The camera has 1000 (H) × 1000 (V) pixels and axial separation between moving image sensor, *Δz*, is 2 mm. We used a 3D car model in Figure 3b as the 3D object. Its location is *z_r_* = 320 mm. Finally, we recorded 50 multiple images using ADS. In this experiment, we use both non-occluded and occluded 3D objects as shown in Figure 3b,c. The photon counting images are obtained digitally by applying the Poisson model (Equations (1) and (2)) to the digitally captured elemental images.

**Figure 4:**
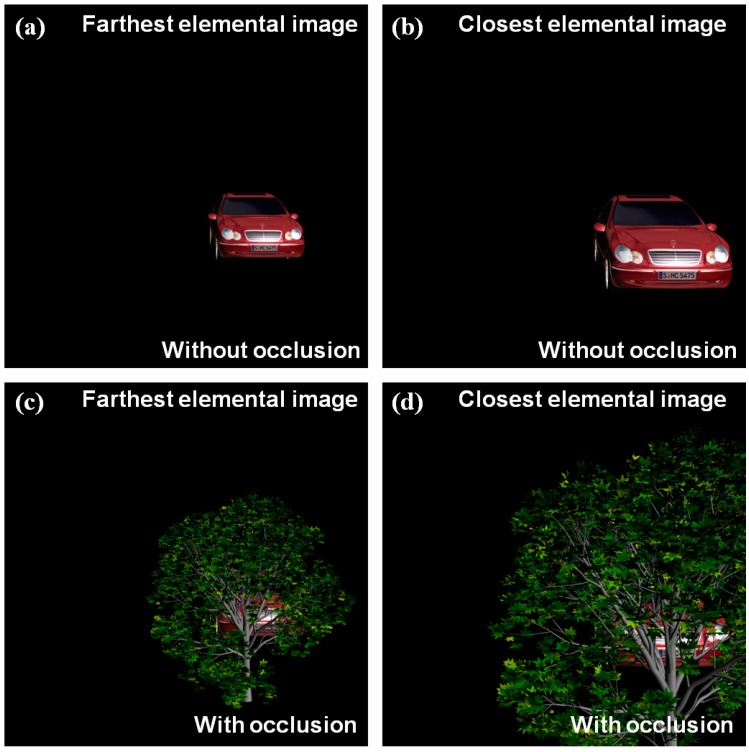
Conventional elemental images with slightly different perspectives by ADS, for: (**a**,**b**) non-occluded object; (**c**,**d**) occluded object, respectively.

Figure 4 shows conventional elemental images by ADS for non-occluded 3D object and occluded 3D object. It is noticed that slightly different perspectives between farthest and closest images exist. Thus, using these perspectives and computational reconstruction of ADS, we can reconstruct 3D images. 

#### 3.1.2. Results

**Figure 5:**
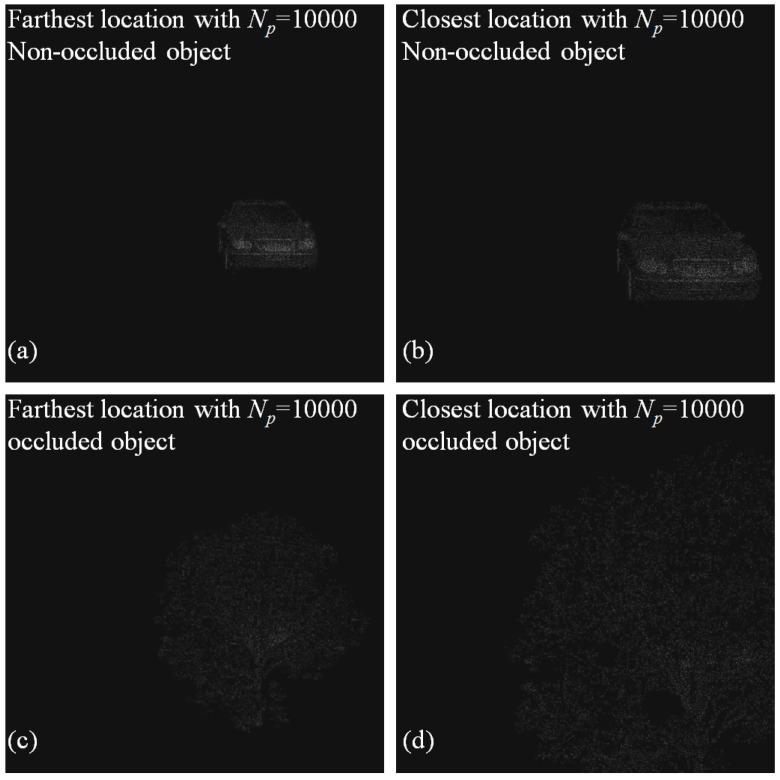
Photon-limited elemental images with *N_p_* = 10,000 of non-occluded object and occluded object at (**a**,**c**) farthest location from camera and (**b**,**d**) closest location from camera.

**Figure 6:**
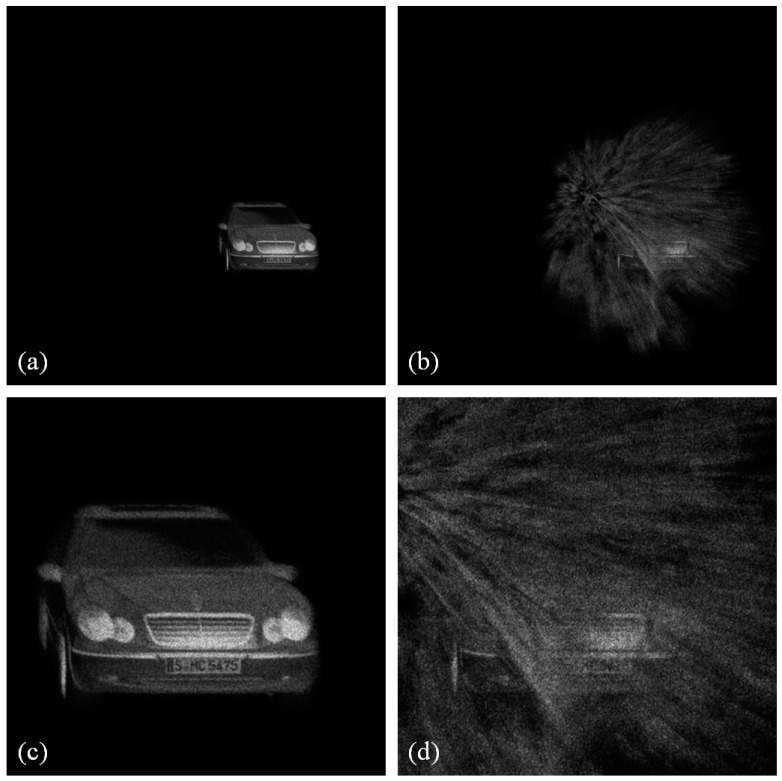
Reconstruction results of photon counting ADS with *N_p_* = 10,000 for (**a**) non-occluded object and (**b**) occluded object. Enlarged part of the reconstructed 3D images for (**c**) non-occluded object and (**d**) occluded object.

**Figure 7:**
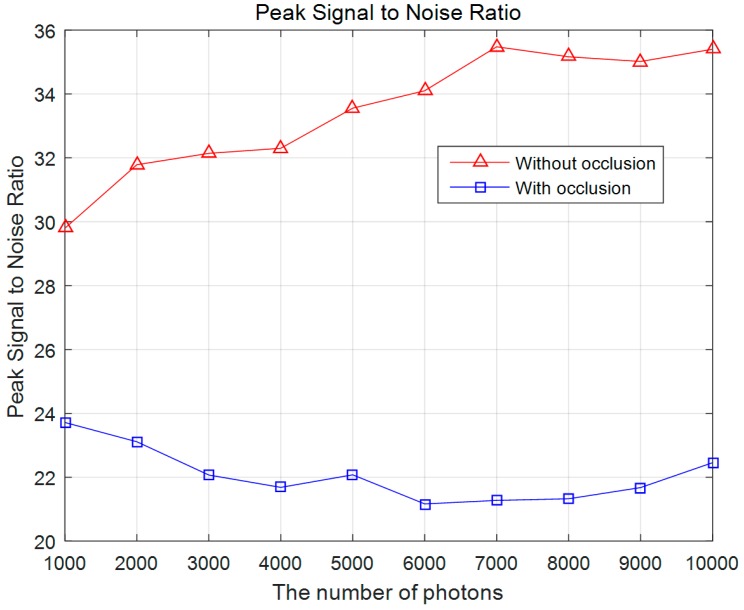
Peak Signal to Noise Ratio (PSNR).

Figure 5 shows photon-limited elemental images for photon counting ADS of non-occluded and occluded objects at farthest and closest positions, which are obtained by using Equations (1) and (2) applied to the digitally captured images. Since the number of photons is low, that is *N_p_* = 10,000 (0.01 photons/pixel), its visual quality is low and the objects are not well recognized. However, using computational reconstruction of ADS, as depicted in Equations (3)–(7), the visual quality of the reconstructed image can be improved, as shown in Figure 6. To evaluate the visual quality of the reconstructed 3D images, we calculate the peak signal to noise ratio (PSNR) between the original reconstructed Three-dimensional images using conventional ADS and the experimental results as shown in Figure 7. The plot has some fluctuations because photons are generated by Poisson random process.

Using multiple images obtained by ADS, we can regenerate the elemental images for 3D multi-view display [18]. Then, the depth map of the 3D objects can be extracted by 3D profilometry [19]. In [19], the extracted depth with contours of equal depth is shown. The errors of the estimated depth may occur due to the specular reflection off of the glossy surface, which departs from the Lambertian assumption. In addition, in [19], the computational reconstruction results of the 3D objects by slicing them in a certain depth range are shown. 

## 4. Conclusions

In this paper, we have presented an overview of 3D photon counting imaging system under photon-starved conditions using ADS. Photon counting ADS uses camera movement along the optical axis unlike photon counting integral imaging which requires lateral parallax. It can reconstruct 3D images under photon starved conditions, including occluded objects. For real-time photon counting ADS, faster reconstruction algorithm with a Graphic Processing Unit (GPU) may be required.

## Abbreviations

The following abbreviations are used in this manuscript:

ADS	Axially distributed sensing
CIIR	Computational integral imaging reconstruction
MLE	Maximum likelihood estimation
SAII	Synthetic aperture integral imaging
